# Retraction Note to: Knowledge, practice and associated factors of newborn care among postnatal mothers at health centers, Bahir Dar City, Northwestern Ethiopia, 2016

**DOI:** 10.1186/s13104-020-05217-9

**Published:** 2020-08-03

**Authors:** Awoke Kebede

**Affiliations:** School of Nursing, College of Health Sciences and Comprehensive Specialized Hospital, Aksum University, Aksum, Ethiopia

## Retraction to: BMC Res Notes (2019) 12:483 10.1186/s13104-019-4501-z

The Editor has retracted this article [1]. An investigation by Aksum University found significant overlap of both text and data with the Master’s Thesis of Zeleke Aschalew Melketsedik, “Knowledge, attitude and practice of newborn care among postnatal mothers at governmental health centers, Addis Ababa, Ethiopia, 2016”, which was defended at the School of Graduate Studies of Addis Ababa University, School of Allied Health Sciences, Department of Nursing and Midwifery, in June 2016.

Awoke Kebede agrees with this retraction.
